# Combined Microwave Pretreatment and MMA Impregnation for the Development of High-Performance Wood–Polymer Composites

**DOI:** 10.3390/polym18101185

**Published:** 2026-05-12

**Authors:** Fernando Júnior Resende Mascarenhas, André Luis Christoforo, Rogério Manuel Santos Simões, Alfredo Manuel Pereira Geraldes Dias, André Eduardo Palos Cunha, Francisco Antonio Rocco Lahr

**Affiliations:** 1 Department of Civil Engineering, University of Coimbra, ISISE, ARISE, Rua Luís Reis Santos—Pólo II, 3030-788 Coimbra, Portugal; alfgdias@dec.uc.pt; 2 Innovation and Competence Forest Centre (SerQ), Rua J, Nº 9, Zona Industrial da Sertã, 6100-711 Sertã, Portugal; 3 Postgraduate Program in Civil Engineering (PPGECiv), Department of Civil Engineering (DECiv), Federal University of São Carlos (UFSCar), Rodovia Washington Luís (SP-11 310), Km 235, São Carlos 13565-905, Brazil; alchristoforo@ufscar.br; 4 Unit of Fiber Materials and Environmental Technologies (FibEnTech), Department of Chemistry, University Beira Interior (UBI), Rua Marquês D’Ávila e Bolama, 6201-001 Covilhã, Portugal; rmss@ubi.pt (R.M.S.S.); aepcunha@gmail.com (A.E.P.C.); 5 Department of Structural Engineering, University of São Paulo, Av. Trab. São Carlense, 400, Parque Arnold Schimidt, São Carlos 13566-590, Brazil; frocco@sc.usp.br

**Keywords:** microwave modification, resin treatment, maritime pine, treatability, water repellence, mechanical performance, bending strength

## Abstract

Wood–polymer composites (WPCs) produced through monomer impregnation have attracted increasing interest as a strategy to improve the durability and performance of wood materials. However, the limited permeability of certain wood species often restricts the effectiveness of impregnation treatments. This study investigates the use of microwave (MW) pretreatment as a drying and microstructural modification step to enhance methyl methacrylate (MMA) impregnation and in situ polymerization in maritime pine (*Pinus pinaster*) heartwood specimens. Wood specimens were subjected to MW treatment of 700 W and 5 min cycles prior to vacuum-pressure impregnation with MMA and subsequent thermal polymerization. Scanning electron microscopy and treatability parameters confirmed that MW pretreatment increased wood impregnability by generating microcracks and improving monomer penetration, thereby resulting in higher polymer retention and a higher weight percentage gain. As a result, the combined MW+MMA treatment produced a more homogeneous distribution of polymethyl methacrylate within the wood structure. The modified specimens showed a substantial reduction in water absorption and the highest water repellence efficiency among the studied groups, while dimensional stability improved to a lesser extent. In addition, the combined treatment significantly increased bending strength and stiffness, indicating an effective reinforcement of the wood structure through polymer loading. These results demonstrate that MW pretreatment is an efficient strategy to improve the treatability of maritime pine heartwood and to enhance the performance of MMA-based WPCs.

## 1. Introduction

The use of wood as a construction material has gained increasing attention, owing to its sustainable nature and appealing physical, mechanical, and aesthetic characteristics [1,2]. Even so, wood as a construction material may present certain issues related to low dimensional stability, limited permeability, poor thermal stability, and reduced biological resistance [3,4,5]. To address these issues, different physical and chemical modification treatments have been investigated and used, including electromagnetic technology and monomer impregnation.

Several studies have demonstrated that combining physical and chemical treatments can yield synergistic effects, improving the overall performance of wood. The integration of chemical impregnation with densification processes has been shown to significantly enhance mechanical properties and dimensional stability [6]. Similarly, the combination of thermal treatment and wax impregnation has proven effective in improving durability and reducing hygroscopicity through synergistic interactions between the two processes [7]. Other approaches have explored multi-step treatments involving thermal pretreatment followed by styrene impregnation and in situ polymerization, leading to improved thermal stability, moisture resistance, and mechanical performance [8]. These findings reflect a broader trend in lignocellulosic modification, where combined treatments are often more effective than individual processes due to complementary mechanisms [9,10].

In the resin impregnation, some monomers have been historically used for wood impregnation, such as styrene (St) and methyl methacrylate (MMA) [8,11,12]. MMA has attracted attention not only for producing wood–polymer composites (WPCs), but also for recent practices in manufacturing transparent wood [13,14,15,16,17,18,19]. In the manufacture of WPCs, the MMA solution is usually impregnated into wood via vacuum-pressure cycles, and the resulting material is then in situ polymerized using heat [19,20]. MMA-derived polymers, such as polymethyl methacrylate (PMMA), are widely used in construction, automotive, and healthcare sectors [21] due to their durability, UV resistance, low weight, good mechanical properties, and biocompatibility [21,22,23]. In the context of wood modification, MMA’s role is particularly relevant, as the formation of a polymer phase within the wood structure contributes to moisture exclusion and mechanical reinforcement [11].

The polymerized MMA provides reduced equilibrium moisture content, improved water repellence, dimensional stability and weathering resistance, greater mechanical properties, and enhanced biological resistance to decay fungi and insects of several wood species [11,19,24,25,26]. For instance, *Pinus elliottii* wood specimens treated with MMA had increases in the bending modulus of rupture (MOR) and modulus of elasticity (MOE) up to 66.4 and 16.8%, respectively, with a reduction in the equilibrium moisture content of 60.0%, and an increase in the thermal stability of the WPCs [27]. These improvements are generally associated with the ability of the polymer to occupy lumens and voids, thereby limiting moisture ingress and reinforcing the internal structure [11,28].

Despite these advantages, the effectiveness of MMA-based treatments is strongly dependent on the permeability of the wood substrate [29,30,31]. MMA impregnation often fails in low-permeability regions, such as heartwood, where anatomical barriers significantly restrict fluid transport [32,33]. Hence, the penetration of monomers and resins is not sufficiently deep or uniform to ensure a consistent modification of the material [29,34], resulting in heterogeneous polymer distribution and suboptimal physical and mechanical properties [35].

Among the available techniques to increase wood permeability, microwave (MW) treatment has gained increasing attention as an effective and environmentally friendly technological process. The interaction between MW energy and the moisture present in wood generates internal vapor pressure, which promotes the formation of microcracks and the opening of the cell wall pores. As a result, the internal structure becomes more accessible to fluids, mainly for refractory wood species. Therefore, it has been widely explored at laboratorial scale to facilitate the penetration of preservatives into regions that are otherwise difficult to treat [19,30,36,37,38].

The effects of MW treatment are strongly dependent on the wood species, their stem part, densities, anatomies, and initial moisture content, as well as on the applied MW treatment parameters [36,39]. Low MW treatment intensity, i.e., low MW power and continuous exposure time, can increase permeability, with no statistically significant reduction in mechanical properties [36]. For instance, when MW-treating *Eucalyptus globulus* heartwood specimens at low intensity, the uptake of a preservative product increased up to 51.6%, while no reductions were reported for different mechanical properties.

Higher MW power levels and longer continuous exposure times generally lead to more pronounced increases in permeability, which is highly beneficial for subsequent impregnation processes [30,36,40]. However, severe treatment conditions may induce excessive microstructural damage, such as cracking or degradation of cell wall integrity, thereby negatively affecting mechanical performance [36]. High-intensity MW treatment caused reductions of up to 59.3% and 36.2% in the bending MOR and MOE, respectively, in *Pinus radiata* heartwood specimens [41]. *E. globulus* heartwood specimens treated with moderate to high-intensity MW conditions presented increases in the preservative retention up to 106.3%, but reductions of 22.2 and 14.8% in the bending MOR and MOE, respectively.

Despite its potential, the application of MW technology has been predominantly investigated as a means to optimize drying kinetics or to increase permeability for the uptake of traditional biocides and preservatives [38,42]. While this focus on biological durability is essential, the use of MW-induced permeability as a gateway for deep resin impregnation remains remarkably under-explored. The viability of this synergy was recently demonstrated in *E. globulus* heartwood specimens, where the combination of MW and MMA resulted in a 63% increase in polymer load and a 38% improvement in dimensional stability [19].

However, these findings cannot be directly extrapolated to softwood species, as fluid transport and permeability in such species are governed by distinct anatomical and chemical factors [43], and each wood species reacts differently to the same MW treatment configuration [38,44]. In this context, the combination of MW pretreatment with MMA impregnation and in situ polymerization warrants further studies on softwood species, particularly maritime pine (*Pinus pinaster*), which is one of the most important softwood species in Southern Europe, including countries such as Portugal, Spain, and Italy [45,46]. It represents a key resource in the regional forest-based economy, with a significant annual timber production that supports well-established industrial value chains [47,48]. Due to its availability and good mechanical performance, this species is widely used in construction-related applications, including structural elements and utility poles [49].

However, it exhibits limited dimensional stability, low natural durability, and is difficult to be treated with preservative products [50]. The heartwood of maritime pine is classified as extremely difficult to treat (Class 4) according to EN 350 [51], limiting the effectiveness of conventional impregnation-based modification processes. Hence, the combination of high structural potential and low treatability makes maritime pine a particularly relevant candidate for the development of advanced modification strategies aimed at enhancing its properties. In this context, it has been a source of studies on other wood modification strategies, including thermal treatment and furfurylation, some of which may involve trade-offs between improved durability and mechanical performance [52,53,54,55].

Unlike refractory hardwoods such as *E. globulus*, where low permeability is mainly associated with vessel occlusion and complex anatomical barriers, the poor treatability of *P. pinaster* heartwood is more closely related to the presence of extractives and resin that obstruct flow pathways. In this context, previous studies with maritime pine specimens showed that MW treatment can promote not only moisture removal but also partial removal and redistribution of resinous components, thereby improving impregnability [30,56]. This difference suggests that the mechanisms by which MW treatment enhances permeability may differ significantly between these species. This distinction is particularly relevant, as it indicates that processing parameters and expected outcomes may not be directly extrapolated from hardwood to softwood species [38,39], reinforcing the need for species-specific investigations.

Based on these considerations, it is hypothesized that MW pretreatment enhances the impregnability of *P. pinaster* heartwood, thereby enabling a more effective penetration and distribution of MMA within the wood structure and ultimately improving the physical and mechanical performance of the resulting WPCs. Therefore, this study aims to investigate an MW treatment as a drying and pretreatment step for the subsequent impregnation and in situ polymerization of MMA to produce high-performance maritime pine heartwood composites. The objective is to evaluate its effectiveness in enhancing impregnability and treatability parameters, and in improving the physical and mechanical properties of the maritime pine wood–polymer specimens so that they can be used as construction materials.

## 2. Materials and Methods

### 2.1. Materials

Small clear maritime pine (*Pinus pinaster* Aiton) heartwood specimens, obtained from the central region of Portugal, were cut to final dimensions of 320 mm in the longitudinal (L) direction and 20 mm in both the radial (R) and tangential (T) directions. These dimensions were selected to ensure compatibility with bending tests and with the MW treatment and impregnation procedures. Within each group, paired specimens were obtained from adjacent regions of the same board whenever possible, in order to minimize variability along the length and ensure a more consistent comparison between treatments.

A total of 64 specimens were produced, of which 32 were subjected to MW treatment, while the remaining 32 were kept untreated (conventional air kiln drying) and used as reference material. In contrast to previous studies that primarily investigated sapwood due to its higher natural impregnability [20,57], the present work focused on heartwood, which represents a more challenging stem part [30].

Within each group of 32 specimens, 16 were further impregnated with MMA and subsequently polymerized in situ. The reference specimens without MW treatment and without MMA impregnation were designated as “REF”. Those impregnated with MMA but not exposed to MW treatment were labeled “REF+MMA”. Samples subjected to only MW treatment were identified as “MW_700”, while specimens that underwent both MW treatment and MMA impregnation were referred to as “MW_700+MMA”.

The monomer used for the impregnation process was MMA (99% purity), supplied by Arpadis (Antwerp, Belgium), and stabilized with hydroquinone monomethyl ether as an inhibitor. MMA was selected for its low viscosity and well-established effectiveness in wood–polymer composite applications, particularly for in situ polymerization and property enhancement. Its Chemical Abstracts Service (CAS) number is 80-62-6, and the corresponding European Community (EC) number is 201-297-1. Benzoyl peroxide (BPO), supplied as a white powder, was used as the thermal initiator for the polymerization reaction.

### 2.2. MW Pretreatment

The maritime pine specimens, with an initial moisture content (IMC) of 71.4 ± 2.5%, were subjected to MW treatment in an oven operating at 2.45 GHz and equipped with a rotating platform of 360 mm. The treatment was continued until an average final moisture content of 12 ± 5% was reached. The MW treatment parameters and the experimental protocol adopted in this study were defined based on the methodology previously established by Mascarenhas et al. [30]. The specimens were arranged in groups of four and exposed to MW at a power of 700 W under continuous exposure time for 5 min. At the end of each 5 min cycle, a 30 s cooling interval was introduced to allow the measurement of the moisture content (MC) of each specimen and to prevent excessive temperature rise [58]. In the present work, four specimens with a total combined volume of 512 cm^3^ were treated simultaneously in the MW oven. Based on this configuration, the specific power was calculated using the approach proposed in [22], yielding 1.37 W/cm^3^.

### 2.3. The Manufacture Process of the WPCs and the Characterization of Monomer Impregnation and in Situ Polymerization

**Figure 1:**
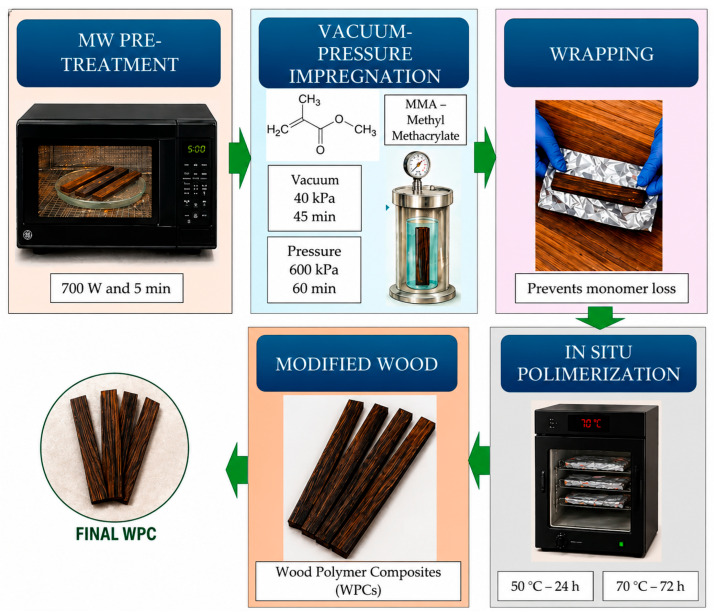
Schematic representation of the wood–polymer composite manufacturing process.

The experimental procedure for treating wood with MMA, including monomer preparation, impregnation, and in situ polymerization, was established based on previously reported methodologies [19,20,27], complemented by preliminary trials conducted in this study (Figure 1). Both reference and MW-treated pine specimens were initially oven-dried at 90 ± 2 °C until constant weight was achieved. This step ensured the complete removal of moisture from the wood. After drying, the samples were allowed to cool to room temperature prior to impregnation.

The monomer solution was prepared by adding 1.75 wt% BPO, the thermal initiator, to MMA, followed by homogenization under magnetic stirring for 30 min. Impregnation was performed in a vertical autoclave with a capacity of 5700 cm^3^. The wood specimens were placed inside the autoclave and first subjected to a vacuum phase at 40 kPa for 45 min, after which a pressure of 600 kPa was applied for 1 h (Figure 1). Upon completion of the impregnation cycle, the specimens were removed, and the excess monomer solution was carefully wiped from their surfaces.

Subsequently, the impregnated specimens were individually wrapped in aluminum foil and subjected to in situ polymerization. This process was carried out in two sequential stages: first in an oven at 50 °C for 24 h, followed by a second stage at 70 °C for 72 h (Figure 1). The first stage was intended to promote the initiation and progression of polymerization, while the second stage contributed to further polymer development and to the removal of residual non-polymerized MMA from the wood structure. Polymerization was carried out in a laboratory oven equipped with a built-in temperature control and ventilation system, ensuring stable, uniform thermal conditions throughout the process.

All impregnation and polymerization procedures were conducted in strict compliance with the safety recommendations provided by the MMA manufacturer. Appropriate personal protective equipment, including safety goggles, a laboratory coat, gloves, and a suitable respirator mask for protection against gases and vapors, was used throughout the process. In addition, the adopted experimental configuration limited airborne emissions, as the MMA remained confined within the wood structure during polymerization, thereby minimizing potential environmental exposure.

The density of the wood specimens, according to ISO 13061-2 [59], was determined at different stages of the experimental procedure, namely before and after the MW treatment and before and after MMA impregnation and polymerization. All density values were subsequently corrected to a reference moisture content of 12% (ρ_12_%). Based on these measurements, the permanent variation in density (PVD), expressed as percentages, was calculated by comparing the density of the specimens before impregnation and after polymerization, according to Equation (1) [19,20].

(1) $$PVD=\frac{\rho_{p}-\rho_{d}}{\rho_{d}}\times 100$$

where $\rho_{p}$ is the density of the polymerized wood specimens, in g/cm^3^; and $\rho_{d}$ is the density of the untreated wood specimens, in g/cm^3^.

The retention of monomer (*Rₘ*) and the retention of polymer (*R_p_*), expressed in g/cm^3^, were determined using Equations (2) and (3), respectively [19,20]. These parameters provide quantitative information on the amount of MMA initially absorbed by the wood and the amount of polymer effectively retained after in situ polymerization.

(2) $$R_{m}=\frac{w_{i}-w_{d}}{v}$$

(3) $$R_{p}=\frac{w_{p}-w_{d}}{v}$$

where $w_{i}$ is the weight of the impregnated pine wood samples, in g; $w_{d}$ is the oven-dried weight of the pine samples, in g; v is the volume of the pine wood samples before impregnation, in cm^3^; and $w_{p}$ is the weight of the polymerized wood pine samples, in g.

The percentage conversion of monomers into polymers (*C*) was calculated according to Equation (4) and was used as an indicator of polymerization efficiency and monomer fixation within the wood structure [28]. It should be noted that this parameter does not represent the absolute chemical conversion of MMA, which would require direct analytical techniques such as infrared spectroscopy or residual monomer quantification. Instead, it should be understood as a gravimetric assessment of the fraction of monomer effectively retained as polymer after the thermal treatment.

(4) $$C=\frac{w_{p}-w_{d}}{w_{i}-w_{d}}\times 100$$

In addition, the weight percentage gain (*WPG*), which represents the weight fraction of polymer relative to the initial dry weight of wood and is commonly associated with polymer loading, was determined using Equation (5). This parameter is closely related to changes in most of the physical, mechanical, and durability-related properties of the resulting WPCs [28].

(5) $$WPG=\frac{w_{p}-w_{d}}{w_{d}}\times 100$$

### 2.4. Microscopic Investigation

Scanning electron microscopy (SEM) was performed to identify the presence of polymerized MMA within the cellular structure. Prior to observation, the samples were oven-dried at 70 °C until reaching constant mass to minimize potential deformation or damage to the wood microstructure. Thin sections suitable for SEM analysis were subsequently prepared using a microtome. The preparation of the wood specimens followed procedures indicated in the literature [20,60]. The prepared specimens were then gold-coated and examined using a Hitachi S-3400N scanning electron microscope (VP SEM Hitachi S-3400N, Tokyo, Japan).

### 2.5. Water Repellence Efficiency (WRE) and Anti-Swelling Efficiency (ASE)

The percentage of water absorption (*WA*) was first determined (Equation (6)) using specimens measuring 30 mm (L) × 20 mm (R) × 20 mm (T), over a four-week period corresponding to four wetting-drying cycles [12]. At the beginning of each weekly cycle (day 1; 0 h), the oven-dry mass and volume of the specimens were measured and recorded. After five days of immersion (96 h), the saturated mass and volume of each specimen were again determined. Subsequently, the specimens were oven-dried at 103 ± 2 °C until reaching constant mass, after which the same procedure was repeated for the remaining cycles up to week four. This protocol constituted the first step in evaluating the hydrophobicity of maritime pine heartwood and its corresponding WPC specimens.

(6) $$WA=\frac{w_{f}-w_{i}}{w_{i}}\times 100$$

where WA denotes the percentage of water absorbed, in %; $w_{f}$ is the specimen mass after 96 h of water exposure, in g; and $w_{i}$ corresponds to the initial mass measured at 0 h, in g.

Based on these measurements, the water repellence efficiency (*WRE*) was calculated as a percentage using Equation (7) [12].

(7) $$WRE=\frac{{WA}_{u}-{WA}_{t}}{{WA}_{u}}\times 100$$

where u refers to the untreated reference specimens (R), and t corresponds to the treated samples (REF+MMA, MW_700, and MW_700+MMA).

The anti-swelling efficiency (*ASE*) was subsequently determined as a percentage according to Equations (8) and (9) [12] using the same specimens measuring 30 mm (L) × 20 mm (R) × 20 mm (T),

(8) $$ASE=\frac{S_{u}-S_{t}}{S_{u}}\times 100$$

(9) $$S=\frac{V_{f}-V_{i}}{V_{i}}\times 100$$

In this expression, *S* represents the swelling percentage, *V_f_* is the specimen volume after 96 h of immersion (cm^3^), and *V_i_* is the initial volume measured at 0 h (cm^3^).

After completing the *WRE* and *ASE* evaluations, the leaching percentage (*L*) was also estimated using Equation (10). This calculation considered the specimen mass recorded at 0 h during the first cycle and the mass measured after 96 h in the fourth cycle.

(10) $$L=\frac{w_{p}-w_{l}}{w_{l}}\times 100$$

where *w_l_* corresponds to the weight of the pine wood and WPC specimens after the leaching process.

### 2.6. Mechanical Properties

Prior to the mechanical testing, all wood specimens were conditioned in a climate-controlled environment until they reached constant weight. Three key mechanical parameters were subsequently determined at a moisture content of 12%: the ultimate bending strength (MOR_12%_), the modulus of elasticity in static bending (MOE_12%_), and the compressive strength parallel to the grain (f_c,0,12%_). The bending tests were conducted using specimens measuring 320 mm (L) × 20 mm (R) × 20 mm (T), in accordance with the procedures described in ISO 13061-3 [61] and ISO 13061-4 [62], respectively. The compressive tests were performed using specimens measuring 60 mm (L) × 20 mm (R) × 20 mm (T), in accordance with the procedures described in ISO 13061-17 [63].

For both bending mechanical tests, loading was applied using a testing actuator with a maximum capacity of 25 kN; for the compressive tests, an actuator with a maximum capacity of 50 kN was used. The resulting displacements in the bending test were monitored using a 25 mm linear variable differential transformer (LVDT), allowing determination of the modulus of elasticity.

### 2.7. Analysis of the Results

Considering the four experimental groups of wood specimens, REF, REF+MMA, MW_700, and MW_700+MMA, a one-way analysis of variance (ANOVA) was conducted using a significance level of 5%. According to the results of the ANOVA followed by Tukey’s post hoc test, the mean values obtained for a given property were considered statistically different among groups when the *p*-values were below the established significance threshold (*p* < 0.05). Prior to performing these analyses, the experimental data were evaluated for normality using the Anderson–Darling test, also adopting a significance level of 5% (*p* > 0.05). All statistical analyses were performed using Minitab software (Version 18) [64].

To better understand the relative influence of the MW and MMA treatments on the physical and mechanical properties of the material, a factorial analysis was performed. In this approach, the effects of MW and MMA treatments were evaluated both individually and in combination.

Pareto charts were used as a graphical tool to represent the standardized effects of each factor and their interaction. This method provides a clear visualization of the relative importance of the variables, enabling identification of the factors with the greatest impact on the response variables. The use of Pareto charts has been widely reported in experimental and optimization studies involving multiple variables, particularly in materials science and process analysis [65,66,67].

The standardized effects were calculated by normalizing the estimated effects with respect to their associated variability, allowing direct comparison between parameters. In the Pareto charts, the magnitude of each effect is represented by horizontal bars, while a reference line indicates the threshold for statistical significance [66].

This type of analysis is commonly employed to identify dominant factors and interactions in multi-parameter systems, supporting a more systematic interpretation of experimental results [68]. In the present study, it was particularly useful for assessing not only the individual contributions of MW and MMA treatments, but also their combined effect on the observed properties, thereby enabling a clearer understanding of the mechanisms governing the material behavior.

It should be noted that no direct characterization of the polymer phase, such as molecular weight distribution or spectroscopic confirmation of chemical structure, was performed in this study. The formation of a polymer phase consistent with PMMA was inferred based on the well-established free-radical polymerization mechanism of MMA in the presence of thermal initiators [11,69]. This approach is consistent with previous studies [19,20,27,70,71] on WPCs, which primarily evaluate treatment efficiency based on macroscopic properties, such as weight percent gain, mechanical performance, and microstructural observations. Therefore, the present study focuses on the technological performance of the modified material rather than on detailed polymer characterization.

## 3. Results and Discussion

### 3.1. Treatability Analysis

**Figure 2:**
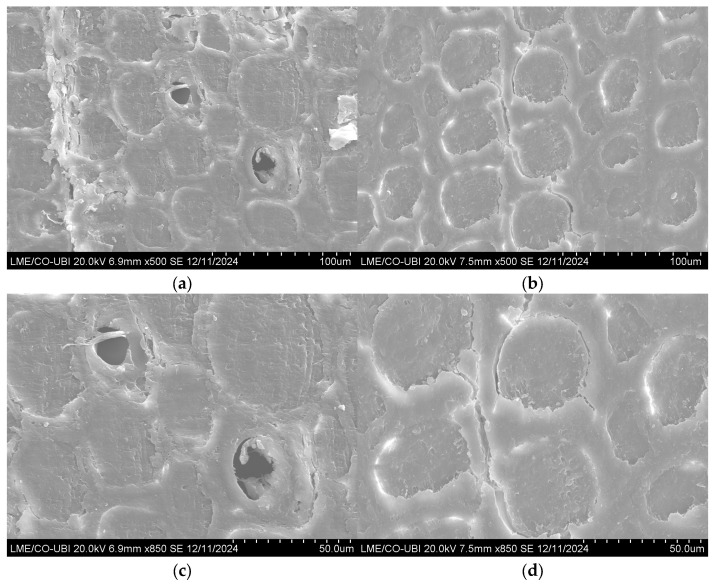
SEM images of the cross-section of the maritime pine heartwood (**a**) REF+MMA and (**b**) MW_700+MMA specimens amplified 500 times, and (**c**) REF+MMA and (**d**) MW_700+MMA amplified 850 times.

Scanning electron microscopy (SEM) provided the first evidence of the distribution of polymerized MMA within the wood cellular structure (Figure 2). In the specimens belonging to the REF+MMA group, the polymer deposits were mainly observed within the natural lumens and pores of the wood tissue. Although the presence of polymer was clearly identified in several cells, some regions remained only partially filled, and empty spaces were still visible inside certain lumens (Figure 2a,c). A comparable microstructural pattern was reported for MMA-modified *Pinus taeda*, where polymer deposition occurred mainly within cell cavities, but did not completely fill the available anatomical spaces [20]. This behavior is consistent with the inherently limited permeability of pine heartwood, where fluid transport is often restricted by pit aspiration and the presence of extractives and resin that obstruct flow pathways [72]. When treating maritime pine heartwood specimens with furfuryl alcohol without any pretreatment, Esteves et al. [55] observed an uneven distribution of the product in the wood microstructure.

A different microstructural pattern was observed in the specimens previously subjected to MW treatment (MW_700+MMA) (Figure 2b,d). In these specimens, polymerized MMA was detected not only within the natural lumens but also within microcracks and fissures formed during MW exposure. These structural discontinuities are typically associated with the internal vapor pressure produced during rapid MW heating, which locally disrupts the cell wall structure and increases the accessibility of the wood matrix to liquids [73]. Similar results have been reported for MW-treated *Eucalyptus* wood, where the formation of cracks and cavities significantly enhanced the permeability and facilitated resin penetration [74]. It is also important to note that during the MW treatment, in addition to water release, some natural resinous material was removed, as previously reported in the literature [56], which might have contributed to opening the tracheids for better monomer penetration.

The presence of polymer within both the natural lumens and the MW-induced fissures resulted in a more homogeneous distribution of the polymer phase throughout the wood structure. In several regions, PMMA appeared to bridge adjacent anatomical features and fill voids that would otherwise remain empty in untreated material. A similar phenomenon was reported for MW-assisted impregnation of WPCs, where the PMMA was observed filling treatment-induced cavities and producing a more continuous internal structure of the refractory *E. globulus* heartwood specimens [19]. These observations provide microscopic support for the hypothesis that MW treatment not only improves access to existing anatomical voids but also creates additional sites where the monomer can accumulate and subsequently polymerize.

These microscopic observations are consistent with the quantitative treatability parameters (Table 1). Clear differences were observed between the specimens treated with MMA without MW pretreatment (REF+MMA) and those subjected to MW exposure prior to MMA treatment (MW_700+MMA).

Before impregnation, the density of the specimens was slightly lower for the MW-treated group (0.601 g/cm^3^) compared with the REF+MMA specimens (0.658 g/cm^3^). After MMA impregnation and in situ polymerization, however, a pronounced increase in density was observed in both groups. The density of the REF+MMA specimens increased to 0.811 g/cm^3^, whereas the MW_700+MMA specimens reached a higher value of 0.910 g/cm^3^. Consequently, the permanent variation in density (PVD) increased markedly from 23.3% to 51.5%, indicating a substantially greater incorporation of polymerized material within the wood structure when MW pretreatment was applied. *P. pinaster* sapwood specimens showed a 36% increase in the density after being treated with 70% furfuryl alcohol mixture [55].

A similar trend was observed for the impregnation and polymerization parameters. The retention of monomer (Rm) increased from 0.265 g/cm^3^ in the REF+MMA group to 0.406 g/cm^3^ in the MW_700+MMA specimens, an increase of 53.2%. The retention of polymerized solids (Rp) went from 0.118 g/cm^3^ to 0.262 g/cm^3^, representing an increase of 136.4%. This behavior indicates that the additional monomer introduced by the MW treatment was not merely temporarily absorbed but was effectively confined within the structure and polymerized in situ. Similar relationships between MW-induced structural modification and increased monomer retention have been reported for WPCs produced with *E. globulus* heartwood and MMA [19].

The magnitude of the increase in weight percentage gain (WPG) is particularly noteworthy. While the REF+MMA specimens reached a WPG of 15.96%, the MW_700+MMA specimens reached 40.3%, indicating a substantial increase of 183.7% in polymer loading within the wood structure. These values fall within the range reported for MMA-based WPC produced from pine sapwood species, where WPG values were approximately 39% [20]. Very close WPG average values of 38% were found for maritime pine sapwood treated with furfuryl alcohol [55]. In this context, the results obtained here suggest that MW pretreatment was effective in overcoming part of the natural treatability limitations of maritime pine heartwood.

The apparent conversion of monomer into polymer also increased markedly, from 40.2% to 63.6%. When the monomer is more uniformly distributed within the internal volume of the wood, as suggested by the SEM observations, the conditions for in situ polymerization become more favorable. It is important to note that the conversion values reported here were obtained from gravimetric measurements and should therefore be interpreted as apparent conversion values rather than as an absolute chemical conversion of MMA. The difference between monomer uptake and final polymer retention may be attributed to factors such as partial volatilization of MMA during the thermal treatment, loss of weakly retained monomer, and incomplete fixation of the impregnated monomer within the wood structure.

When comparing the present results for *Pinus pinaster* heartwood specimens with those previously reported for *Eucalyptus globulus* under similar MW conditions (700 W and 5 min), important differences can be observed in the role of MW treatment and the efficiency of the impregnation process [19]. Notably, the untreated *P. pinaster* specimens already exhibited Rm and WPG values comparable to those obtained for *E. globulus* after MW treatment, highlighting the inherently higher permeability of the softwood system. In both species, MW pretreatment significantly enhanced monomer uptake and polymer retention; however, in *E. globulus*, a highly refractory hardwood, MW was essential to overcome severe permeability limitations, whereas in *P. pinaster,* it acted primarily as an enhancer of permeability. This distinction is reflected in the results, where the MW_700+MMA group in *P. pinaster* showed an increase in Rm from 0.265 to 0.406 g/cm^3^ and in WPG from 15.96% to 40.28%, while *E. globulus* specimens showed lower absolute values (Rm from 0.158 to 0.248 g/cm^3^ and WPG from 12.24% to 23.94%). Overall, while both species benefit from the combined treatment, the results indicate that in softwoods such as *P. pinaster*, MW treatment plays a key role in optimizing permeability and polymer distribution, rather than enabling impregnation as observed in highly refractory hardwoods.

Interestingly, the leaching values remained very low and statistically equivalent for both groups (approximately 2%), despite the much higher polymer loading in the MW-treated specimens. This suggests that the polymerized MMA remained well retained within the wood structure and was not easily removed during the leaching test. A similar behavior was reported for *Pinus elliottii* wood modified by in situ polymerization with styrene solution, where low leaching values were interpreted as evidence of stable retention of the polymer phase within the cellular structure [8]. In addition, it has been suggested that a portion of the weight lost during leaching tests may correspond to soluble wood components rather than solely to the impregnated polymer.

Although SEM observations confirmed the presence of polymer within cell lumens and microcracks, no quantitative assessment of polymer distribution across the cross-section was performed in this study. Nevertheless, similar approaches have been widely reported in the literature on WPCs, where SEM images are commonly used to qualitatively assess the presence and distribution of polymer within the wood structure [20,27,75]. In this context, the observed microstructural features, together with the relatively high WPG values, suggest that monomer penetration was not restricted to surface regions. However, local variations in polymer distribution across the cross-section cannot be excluded.

### 3.2. Hygroscopicity and Dimensional Stability Analysis

**Figure 3:**
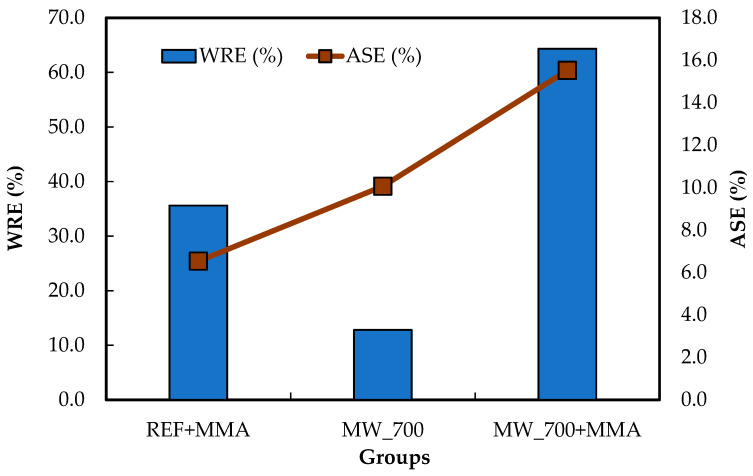
Results of the average water repellence efficiency (WRE) and anti-swelling efficiency (ASE) of the studied wood groups.

The results for water repellence efficiency (WRE) and anti-swelling efficiency (ASE) are presented in Figure 3. The average WRE across the three groups was statistically different, and the average ASE of MW_700+MMA was the only one that differed significantly from the others. As expected, the untreated reference specimens showed the highest water absorption (62.8%) and the highest swelling (9.3%), reflecting the inherently hydrophilic nature of wood. In contrast, all treatments reduced water uptake to some extent, although their effects on dimensional stability were not equally pronounced.

The impregnation of untreated wood with MMA (REF+MMA) reduced WA to 40.5%, corresponding to a WRE of 35.6%. This result indicates that even without MW pretreatment, the polymerized MMA was able to restrict part of the water-accessible volume of the wood. This behavior is consistent with the classical role of polymerized MMA in WPCs, where the polymer primarily occupies lumens and pores and acts as a physical barrier to the penetration of liquid water [12]. However, the reduction in swelling was comparatively modest, with S decreasing only from 9.3% to 8.7% and ASE reaching 6.5%. This contrast is important because it suggests that, in the REF+MMA specimens, MMA acted primarily by reducing water ingress rather than by substantially stabilizing the cell wall itself. A similar distinction between reduced water uptake and only moderate dimensional stabilization has been discussed by Mattos et al. [12] and Acosta et al. [8], who noted that hydrophobicity and lower water absorption do not necessarily translate into strong ASE when the polymer remains concentrated mainly in lumens and capillaries rather than interacting effectively with the cell wall.

The effect of MW treatment alone was more limited in terms of water resistance. The MW_700 specimens still absorbed 54.8% water, corresponding to a WRE of only 12.8%, although swelling decreased to 8.4% and ASE reached 10.1%. Similar results were reported in the literature for different wood species [19,37]. A similar pattern was reported for MW-treated *Eucalyptus*, where MW exposure increased porosity and facilitated water ingress, while the volumetric response remained comparatively lower due to structural changes induced by treatment [19]. Different studies associated this behavior with permanent alterations in the wood structure, including hornification and local changes in cell wall response to moisture (chemical composition) [30,37,76].

The combined treatment (MW_700+MMA) produced by far the best overall performance, reducing WA to 22.4% and swelling to 7.9%, which corresponded to a WRE of 64.3% and an ASE of 15.5%. These results clearly indicate a synergistic interaction between MW pretreatment and MMA impregnation. The SEM observations discussed previously helped explain this behavior: MW treatment generated fissures and additional transport pathways, which facilitated deeper monomer penetration, while subsequent in situ polymerization allowed MMA to occupy not only the natural lumens but also MW-induced discontinuities. As a result, the internal structure became more effectively sealed against the transport of liquid water, which explains the sharp decrease in WA. Similar mechanisms were described by Zhang et al. [37], who reported that MW pretreatment created crack cavities and new flow channels, thereby markedly improving resin infiltration and internal filling. Likewise, Wu and Song [77] showed that MW-assisted impregnation with oxidized paraffin emulsion significantly reduced long-term water absorption because the modifier occupied cell cavities and newly formed cracks, thereby reducing the water-accessible volume of the wood.

**Figure 4:**
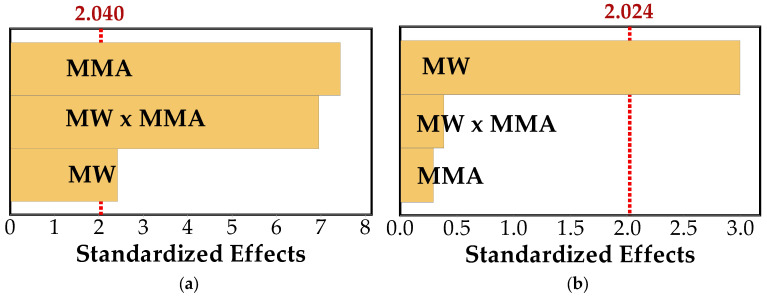
Pareto charts of standardized effects for (**a**) WA and (**b**) S at a significance level of α = 0.05.

A factorial analysis was conducted to better quantify the relative influence of the studied parameters. The results, presented in Figure 4, revealed distinct yet complementary trends for water absorption and swelling.

For WA (Figure 4a), MMA clearly dominated the response, leading to a substantial reduction in water uptake. MW treatment also contributed to this effect, although to a lesser extent. More importantly, the interaction between MW and MMA was pronounced, indicating a synergistic effect in which MW treatment enhanced the effectiveness of MMA. This supports the idea that MW exposure improves the accessibility of the wood structure, facilitating a more efficient penetration and distribution of the monomer and, consequently, limiting moisture uptake.

In contrast, the response observed for S (Figure 4b) followed a different pattern. MW treatment emerged as the most influential factor, promoting a consistent reduction in swelling, while the effect of MMA was comparatively limited. The interaction between both factors was weak, indicating that dimensional stability is primarily governed by structural modifications induced by MW exposure rather than by polymer incorporation.

Taken together, these results reinforce the idea that moisture-related properties are controlled by distinct mechanisms: water absorption is largely governed by the presence and distribution of the polymer phase, whereas dimensional stability is more closely associated with structural changes in the wood matrix.

### 3.3. Evaluation of MOR, MOE, and F_c,0_

**Figure 5:**
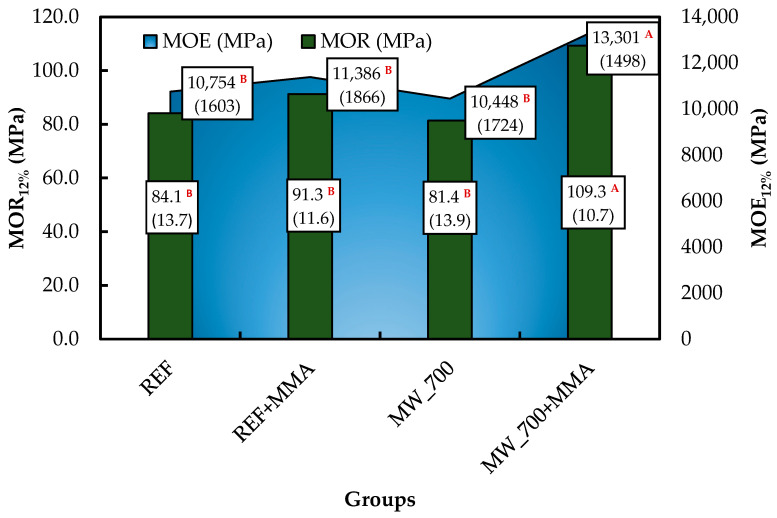
Results of the studies on the bending modulus of rupture (MOR) and modulus of elasticity (MOE) of the studied wood groups. According to Tukey’s test, values identified by different letters indicate statistically significant differences at the 5% significance level (α = 0.05). Values presented in parentheses correspond to the standard deviation.

The bending performance of the specimens is presented in Figure 5. When interpreted together with the previously discussed treatability results, a clear relationship emerges between impregnation efficiency and mechanical behavior, where the bending MOR_12%_ and MOE_12%_ of the MW_700+MMA were statistically different from the others.

The untreated specimens (REF) exhibited a MOR of 84.1 MPa and an MOE of 10,754 MPa, values that are consistent with the expected bending properties of clear maritime pine heartwood specimens [30,78]. These values serve as a useful reference to evaluate the influence of both MW exposure and MMA impregnation.

The first observation concerns the effect of MW treatment alone. The group treated at 700 W and 5 min showed decreases in both MOR (81.4 MPa) and MOE (10,448 MPa) compared with the REF specimens. Although the reduction is modest, it indicates that MW exposure did not directly improve bending performance when applied without subsequent impregnation. This behavior is not unexpected, since MW heating generates internal vapor pressure that may disrupt the cellular structure, producing localized microcracks and increasing permeability while reducing mechanical properties. Similar effects have been reported in studies dealing with MW-treated wood, where increased treatability is sometimes accompanied by a slight reduction in mechanical performance due to microstructural damage when no reinforcing agent is subsequently introduced [30,36,39].

A different trend was observed for the specimens impregnated with MMA without MW pretreatment (REF+MMA). In this case, MOR increased by 8.6% from 84.1 MPa to 91.3 MPa, and MOE increased by 5.9% from 10,754 MPa to 11,386 MPa. Although the improvement was small, it indicates that MMA polymerization inside the wood structure provided some degree of mechanical reinforcement even under limited natural permeability. This effect has been described in WPC specimens based on MMA [20]. Similar increases in bending properties were also reported by Ding et al. [79] for MMA-hardened *Populus* spp. wood, with gains of up to 9.5% in MOE and nearly 17.0% in MOR, depending on the polymer loading.

The most significant improvement was obtained for the combined treatment (MW_700+MMA). In this case, MOR reached 109.3 MPa, and MOE reached 13,301 MPa, representing increases of approximately 30% and 24%, respectively, compared with the REF; 20% and 17% compared with REF+MMA; and 34% and 27% compared with MW_700. Such a pronounced improvement suggests that MW pretreatment played a decisive role in enhancing the reinforcing effect of MMA impregnation. A comparable mechanism has been reported by Mascarenhas et al. [19] for MW-assisted MMA treatment of *E. globulus* heartwood specimens, where the best bending performance was also obtained when MW exposure was followed by MMA impregnation and polymerization.

This interpretation is strongly supported by the treatability parameters discussed earlier. The MW+MMA specimens showed the highest values of Rm, Rp, WPG, conversion, and PVD, as well as a more homogeneous distribution of polymer observed in the SEM images. Once polymerized, the PMMA phase likely filled not only the natural lumens of the wood but also the microfissures generated during microwave treatment. Mattos et al. [20] emphasized that the mechanical response of MMA-based WPCs is closely related to the amount and distribution of polymer retained in the wood.

**Figure 6:**
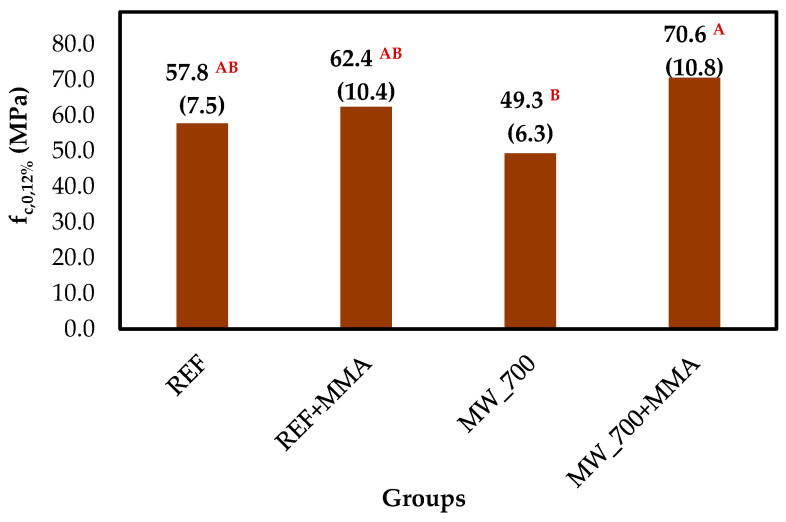
Results of the studies on compressive strength parallel to the grain (f_c,0_) of the studied wood groups. According to Tukey’s test, values identified by different letters indicate statistically significant differences at the 5% significance level (α = 0.05). Values presented in parentheses correspond to the standard deviation.

The compressive strength parallel to the grain at 12% MC (f_c,0,12%_) of the studied groups is presented in Figure 6. The untreated specimens (REF) showed an average value of 57.8 MPa, consistent with typical values reported for maritime pine heartwood [47,78].

A slight 8.0% increase was observed in the REF+MMA group (62.4 MPa), indicating that polymer incorporation contributed to some reinforcement, even under limited permeability conditions. In contrast, the MW_700 group showed a reduction in compressive strength (49.3 MPa), most likely associated with microstructural damage induced by MW treatment, such as the formation of microcracks.

The highest value was obtained for the combined treatment (MW_700+MMA), reaching 70.6 MPa. This corresponds to increases of approximately 22.1% relative to REF and 43.2% relative to MW_700, confirming that MW pretreatment enhances the effectiveness of MMA impregnation and improves mechanical performance. Yildiz et al. [80] treated *P. pinaster* wood specimens with MMA and reported an average increase of 19.3% in the compressive strength parallel to the grain. Ding et al. [79] treated hybrid *Populus* spp. wood specimens with MMA and reported an average increase of 33.3%.

It is also worth noting that the simultaneous increase in MOR, MOE, and f_c,0_ observed in the MW_700+MMA group indicates that the treatment improved not only the ultimate bending strength but also the stiffness of the material. This suggests that the polymer phase contributed to reinforcing the internal structure rather than merely increasing density locally. When the polymer distribution is homogeneous and well-integrated within the wood structure, it can effectively reduce local deformation and delay the onset of failure during bending. From a structural perspective, the results suggest that the MW+MMA treatment did not simply densify the material but created a reinforced WPC in which the polymer contributes to stress transfer within the structure.

**Figure 7:**
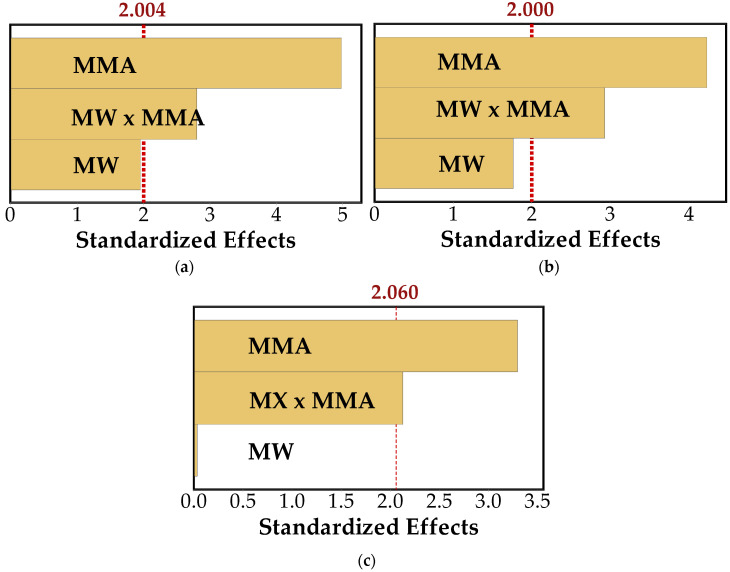
Pareto charts of standardized effects for (**a**) MOR, (**b**) MOE, and (**c**) compressive strength parallel to the grain (f_c,0_) at a significance level of α = 0.05.

To further support this interpretation and better understand the relative influence of the studied factors on mechanical performance, a factorial analysis was conducted, and Pareto charts were used to identify the most significant variables affecting the observed behavior, as shown in Figure 7.

The analysis of standardized effects revealed a consistent pattern across all mechanical properties evaluated. In all cases, MMA emerged as the dominant parameter, with the highest statistical significance and confirming its central role in improving the mechanical performance of the material.

For MOR (Figure 7a), the influence of MMA was particularly pronounced, indicating a clear enhancement in bending strength. Although MW treatment alone did not reach the statistical significance threshold, its interaction with MMA was significant. This suggests that the effectiveness of the polymer treatment depends strongly on prior modification of the wood structure. In this context, MW exposure appears to facilitate a more efficient impregnation of MMA, contributing to a more effective reinforcement mechanism.

A comparable response was observed for MOE (Figure 7b). The stiffness of the material was mainly governed by the presence of MMA, while the isolated effect of MW treatment remained negligible. However, the interaction between the two factors was again significant, reinforcing the idea that MW treatment plays an indirect yet essential role by improving conditions for polymer incorporation. The consistency between MOR and MOE responses further supports this interpretation and points to a common underlying mechanism.

For compressive strength parallel to the grain (Figure 7c), the same general behavior was observed. MMA remained the dominant factor, while the interaction effect (MW × MMA) also reached statistical significance. In contrast, the isolated effect of MW treatment was negligible. This supports the interpretation that MW treatment did not directly contribute to strength development but instead acts as a structural modifier that enhances the accessibility of the wood matrix to the monomer. The resulting improvement in polymer distribution likely leads to more efficient stress transfer under compressive loading.

To further contextualize the results of this study, Table 2 compares the values obtained with those reported in the literature for different wood modification approaches. Overall, treatments based on MMA and other polymer systems tend to improve mechanical properties, though the magnitude of these gains varies with wood species, modification strategy, and processing conditions.

The results obtained for the MW_700+MMA treatment fall within the upper range of values reported for *Pinus* species, particularly when compared to treatments based on MMA without pre-modification, which generally show more modest improvements. This highlights the role of MW pretreatment in enhancing impregnation efficiency and, consequently, mechanical performance.

While some studies report higher increases, these are often associated with more complex formulations, such as multi-component systems or resin-based treatments. From this perspective, the results of the present study show that the combined MW+MMA approach can achieve competitive performance with a relatively simple and effective modification strategy.

## 4. Conclusions

This study investigated the potential of microwave (MW) pretreatment, using 700 W of power and 5 min cycles of continuous exposure time, combined with methyl methacrylate (MMA) impregnation and in situ polymerization to improve the treatability and performance of maritime pine heartwood and to produce high-performance wood–polymer composites (WPCs).

The results demonstrated that the low natural permeability of the heartwood significantly limits impregnation efficiency, leading to reduced monomer uptake and heterogeneous modification when no pretreatment is applied. In this context, MW pretreatment proved an effective strategy to improve wood accessibility by inducing microstructural changes, including the formation of microcracks and the partial removal of resinous components, thereby facilitating fluid transport within the wood structure.

These modifications resulted in a substantial increase in treatability parameters, including monomer uptake, polymer retention, and weight percentage gain, and were further supported by SEM observations indicating more effective luminal filling and treatment-induced cracks. As a result, the MW+MMA treatment produced a more efficient modification of the material.

The improved treatability translated directly into enhanced performance. The combined treatment led to a significant reduction in water absorption and a marked increase in water repellence efficiency, while dimensional stability showed more moderate improvements. In addition, the MW+MMA specimens exhibited the highest mechanical performance, with substantial increases in bending strength, stiffness, and compressive strength. These results indicate that the polymer phase effectively reinforced the wood structure when sufficient penetration was achieved.

The factorial analysis further confirmed that MMA was the dominant factor governing the mechanical response, while MW treatment played a critical enabling role by improving the conditions for polymer incorporation. This highlights the synergistic nature of the combined treatment, in which MW primarily acts as a permeability enhancer, while the MMA polymer provides the main reinforcement effect.

Overall, the results support the initial hypothesis that MW pretreatment enhances the permeability of *P. pinaster* heartwood, thereby promoting higher monomer uptake and polymer retention, which, in turn, leads to improved physical and mechanical performance of the resulting WPCs. Hence, from a practical perspective, the combined MW+MMA approach represents a promising strategy for upgrading difficult-to-treat softwood species, contributing to their potential use in more demanding construction applications.

Future work should focus on evaluating the long-term performance of the modified material under environmental exposure conditions (weathering), as well as on complementary characterization of the polymer phase and wood–polymer interactions using advanced analytical techniques.

## Figures and Tables

**Table 1:** Treatability parameters.

Group	Density 12% Before MMA Treatment (g/cm^3^)	Density 12% After MMA Treatment (g/cm^3^)	PVD (%)	Rm (g/cm^3^)	Rp (g/cm^3^)	WPG (%)	C (%)	L (%)
REF+MMA	0.658 ^A^ (0.054)	0.811 ^B^ (0.102)	23.32 ^B^ (2.54)	0.265 ^B^ (0.054)	0.118 ^B^ (0.021)	15.96 ^B^ (1.85)	40.21 ^B^ (5.01)	2.14 ^A^ (0.09)
MW_700+MMA	0.601 ^B^ (0.042)	0.910 ^A^ (0.036)	51.49 ^A^ (5.32)	0.406 ^A^ (0.039)	0.262 ^A^ (0.059)	40.28 ^A^ (4.07)	63.58 ^A^ (7.25)	2.01 ^A^ (0.07)

According to Tukey’s test, values identified by different letters indicate statistically significant differences at the 5% significance level (α = 0.05). Values presented in parentheses correspond to the standard deviation. PVD = Permanent variation in density. Rm = Retention of monomers. Rp = Retention of polymerized solids. C = Conversion rate. WPG = Weight percentage gain. L = Leaching.

**Table 2:** Comparative analysis of mechanical property variation (MOR, MOE, and f_c,0_) for different wood modification approaches, expressed as percentage relative to untreated wood.

	Variation in a Mechanical Property Compared to Untreated Specimens (%)		
Modification Method	MOR	MOE	f_c,0_
MW_700+MMA [This work]	30.0	23.7	22.1
*P. pinaster* + MMA [80]	6.1	8.3	19.3
*P. pinaster* + Styrene (St) [80]	11.3	12.4	27.5
*P. pinaster* + MMA/St [80]	27.0	31.0	45.8
*Populus x. euramericana* + MMA [80]	38.7	22.6	55.0
*Populus x. euramericana* + St [80]	32.2	23.3	42.1
*Populus x. euramericana* + MMA/St [80]	42.6	22.4	65.8
*P. pinaster* sapwood + Furfuryl Alcohol (FA) [55]	6.0	−0.8	-
*Pinus sylvestris* + Boric Acid (BA) [81]	−14.5	−6.9	-
*Fagus orientalis* + BA [81]	−17.3	−11.7	-
*Pinus taeda* + MMA [20]	22.6	17.1	-
*Pinus taeda* + Glycidyl Methacrylate (GMA) + MMA—3:1 [20]	40.7	20.2	-
*Pinus taeda* + Methacrylic Acid (MAA) + MMA—3:1 [20]	27.3	31.3	-
*Anthochepalus cadamba* + Merbau extractives [82]	3.3	−1.0	-
*Anthochepalus cadamba* + PME22 [82]	28.5	23.5	-
*Anthochepalus cadamba* + PME33 [82]	41.2	40.1	-
*Pinus elliottii* + Thermal [8]	−15.3	−35.1	-
*Pinus elliottii* + St [8]	12.5	−2.1	-
*Pinus elliottii* + Thermal + St [8]	26.4	−16.5	-
*Cunninghamia lanceolata* + FA at 35% [83]	39.2	21.4	-
*Pinus elliottii* + Resin Dissolved in Ethanol (4:1) [84]	37.6	46.6	-
*Pinus elliottii* + Pine Resin Dissolved in Ethanol (4:1) + St (90:10) + Catalyst [84]	43.3	55.9	-
*Pinus elliottii* + Pine Resin Dissolved in Ethanol + St (80:20) + Catalyst [84]	37.4	68.7	-
*Populus deltoides* + Citric Acid + Glicerol [85]	31.7	68.8	56.6

## Data Availability

The data used in the tables and figures in this study are all provided within the text.
